# MEMO1–ERα interaction may occur independent of Y537 phosphorylation which differs from MEMO1-ErbB2 binding

**DOI:** 10.17912/micropub.biology.002363

**Published:** 2026-09-29

**Authors:** Paxton Mills, Haley Salus, Khanh Vu, Lauren Shin, Catherine A Bayas, Joshua Pandian, Carol A. Parish, Julie A Pollock

**Affiliations:** 1 Chemistry Department, University of Richmond, Richmond, VA USA

## Abstract

MEMO1 is a protein implicated in cancer-associated signaling through interactions with other proteins, including estrogen receptor α (ERα). We used fluorescence polarization, molecular dynamics simulations, and protein pull-down assays to characterize the interaction between MEMO1 and ERα. MEMO1 bound to an ERα peptide containing phosphorylated Y537 but unexpectedly showed comparable affinity for the non-phosphorylated peptide. Computational modeling identified distinct binding modes and suggested contributions from MEMO1 residues. Pull-down experiments confirmed interaction between MEMO1 and the ERα ligand-binding domain. These findings suggest that MEMO1 interacts with ERα in a phosphorylation-independent manner, highlighting diverse binding modes that may support its scaffolding functions.

**Figure 1. Examination of interaction of MEMO1 protein with peptides derived from ERα and SRC:** (A) Representative FP binding curve of FL-ERpY537 (0.1 μM, blue) and FL-ERY527 (0.1 μM, orange) with wild type MEMO1 (0 – 4.5 μM) in sodium phosphate buffer (pH 6.4). (B) Representative FP binding curve of FL-SRCpY419 (0.1 μM, light blue) and FL-SRCY419 (0.1 μM, red) with wild type MEMO1 (0 – 4.5 μM) in sodium phosphate buffer (pH 6.4). (C) Displacement of FL-ErbB2pY1222 by unlabeled peptides in sodium phosphate buffer (pH 6.4). (D) Binding affinity of MEMO1 WT and mutants with ERα-derived peptides. Average data points from at least triplicate experiments (± standard deviation) were fit with nonlinear regression to give K _D_ values. (E) A comparison of ERY537 (top) and ERpY537 (bottom) peptide interactions with MEMO1. Structures shown are representative trajectory snapshots with the smallest RMSD from the overall average structure. MEMO1 protein is shown in gray scale, interacting peptide residues are highlighted in blue while atomistic details and distances are included for interacting MEMO1 residues. In the bottom image, the phosphorylated Y is show in red. (F) SDS/PAGE gel of pull-down experiment between His6x-ERα-LBD and MEMO1. Lane 1 = MEMO1; lane 2 = His6x-ERα-LBD; lane 3 = flowthrough; lane 4-6 = wash; lane 7-9 = elution.

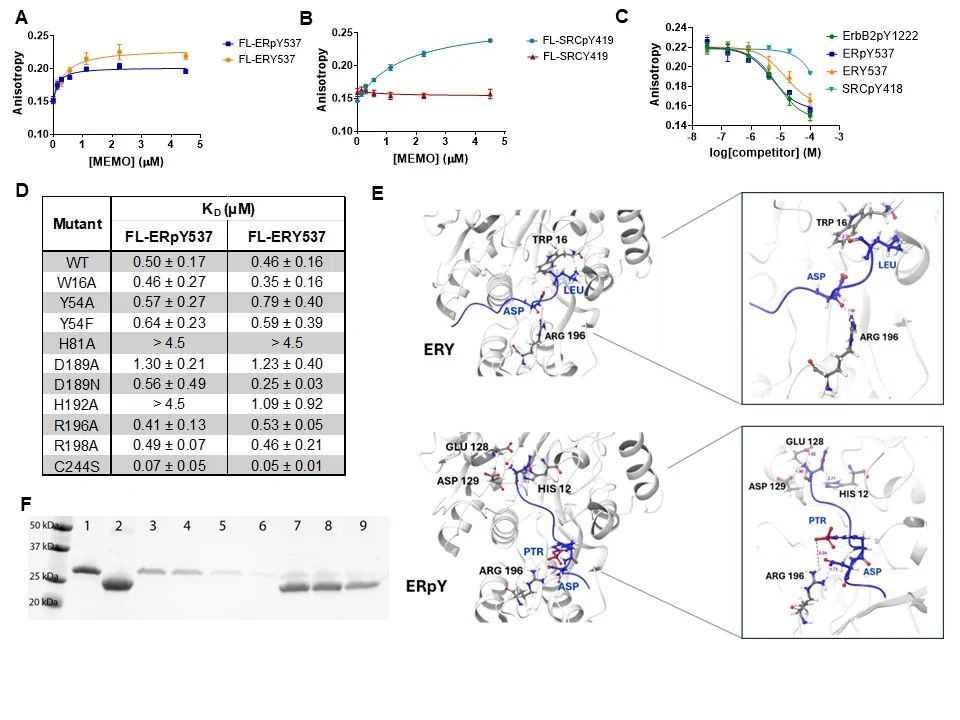



## Description

MEMO1 (Mediator of cell motility 1) is a scaffolding protein that has been shown to interact with phosphorylated ErbB2 to drive migration and aggression of breast cancer tumors (MacDonald et al., 2014; Marone et al., 2004; Qiu et al., 2008). Since the discovery of MEMO1 in 2004, many groups have isolated MEMO1 in protein complexes associated with cancer, including fibroblast growth factor receptor (FGFR), insulin receptor substrate 1 (IRS1), and estrogen receptor alpha (ERα) (Frei et al., 2016; Haenzi et al., 2014; Jiang et al., 2013; Sorokin & Chen, 2013). The molecular interactions of MEMO1 with these other proteins have not been fully elucidated. Previously, we developed a fluorescence polarization (FP) assay to biochemically characterize the interaction between MEMO1 and a fluorescently labeled peptide corresponding to the tail of ErbB2 phosphorylated on Y1222 (Newkirk et al., 2018). More recently, we sought to expand the FP assay to additional potential binding partners of MEMO1 focusing first on ERα. Previous literature has shown that upon heregulin and estradiol activation, MEMO1 acts as a scaffold protein bringing together c-Src kinase (SRC) and ERα to induce phosphorylation of SRC at Y418 and ERα at Y537 (Frei et al., 2016). Downstream consequences of this protein complex include cancer cell proliferation, migration, and endocrine resistance.

To learn more about the interactions of MEMO1 with ERα and SRC at a molecular level, we designed fluorescein-labeled peptides containing the key phosphorylation sites on ERα (Y537) and SRC (Y418) implicated in the cellular study; the phosphorylation sites were confirmed and peptides designed using PhosphositePlus (Hornbeck PV, 2015). Using our FP assay, we examined the binding of purified MEMO1 with the phosphorylated peptides as well as their non-phosphorylated counterparts. We found strong affinity between MEMO1 and the FL-ERpY537 (K _D_ = 0.50 ± 0.17 μM) ( **Figure 1A** ) comparable to our previous findings with the peptide corresponding to ErbB2 (K _D_ = 0.53 ± 0.09 μM) (Newkirk et al., 2018). The interaction between FL-SRCpY419 peptide was weaker (K _D_ = 3.11 ± 2.21 μM) and completely diminished when the phosphate group was removed ( **Figure 1B** ). Most surprising to us was that the non-phosphorylated peptide corresponding to ERα had a similar binding affinity (K _D_ = 0.46 ± 0.16 μM) to the phosphorylated peptide ( **Figure 1A** ).

To investigate the binding information obtained from the initial fluorescence polarization experiments, MEMO1 was incubated with FL-ErbB2pY1222 and titrated with serial dilutions of unlabeled peptides corresponding to ErbB2pY1222, ERpY537, ERY537, and SRCpY418 ( **Figure 1C** ). As expected, the SRC peptide was unable to outcompete the FL-ErbB2pY1222 confirming that the interaction is not as strong. In addition, both ERpY537 and ERY537 were able to displace the ErbB2 peptide with similar K _I_ to the ErBB2pY1222 unlabeled peptide.

Next, we utilized MEMO1 mutants that we previously designed to assess the binding affinities with the ERα derived peptides (Newkirk et al., 2018). As seen in **Figure 1D** , W16A, Y54A, Y54F, D189N, R196A, and R198A had similar binding affinities to the wild-type protein with FL-ERpY537. However, there was decreased binding with D189A, little to no binding observed with H81A and H192A and increased binding affinity with C244S. With the FL-ERY537 peptide, MEMO1 mutants W16A, Y54A, Y54F, D189N, R196A, and R198A exhibited similar binding affinities as the wild-type protein while H192A and D189A showed decreased binding, H81A showed little to no binding, and C244S showed increased binding. These results are not parallel to our previous studies on the ErbB2 derived peptide where mutations of W16A, Y54A, D189A, and R196A significantly diminished binding, Y54F and R198A decreased binding by about half, and H81A, D189N, H192A, and C244S did not have significant impact on the strength of the interaction (Newkirk et al., 2018). Taken together, these data indicate that the binding interaction between MEMO1 and ERα may be different than that of the ErbB2.

To investigate further, we modeled the interactions *in silico* ( **Figure 1E** ). Our MM-GBSA estimates of binding affinity suggest that ERpY537 may bind approximately 10 kcal/mol more tightly to MEMO1 than ERY537. Analysis of the resulting MD trajectories indicates that both the phosphorylated and non-phosphorylated peptides bind fairly similarly; however, the non-phosphorylated peptide spends significantly more time sampling other sites. For instance, MM-GBSA results indicate that binding of the phosphorylated peptide to the illustrated location ( **Figure 1E** , bottom) is significantly favored (–38 kcal/mol) over binding to other sites (–14 and –17 kcal/mol). Binding of the non-phosphorylated peptide occurs in 4 different parts of MEMO1 all of which see affinities of –21 to –29 (one illustrated in **Figure 1E** , top). An analysis of the pairwise contributions to affinity tells us which residue–ligand interactions are strongest. Importantly, in the non-phosphorylated simulations the ERY537–MEMO interactions are few and relatively insignificant whereas in the phosphorylated simulations the pY537 interactions dominate, especially with MEMO1 residues R196, E128, D129, H12 and R198. When comparing the computational modeling to the biochemical results, we postulate that perhaps R196 and R198 can substitute in stabilizing the phosphorylated tyrosine, and this may be why the experimental binding affinity did not change with the individual mutants. In future, we could investigate mutations of E128 and D129. We have been unable to look at H12A because the mutant protein will not fold appropriately (Newkirk et al., 2018).

The most interesting piece of data collected through our biochemical FP assay was the comparable binding of the non-phosphorylated ERα peptide to the phosphorylated peptide. As our previous work and that of others had postulated that MEMO1 is a phosphotyrosine scaffolding protein, we were perplexed by this finding. Therefore, we set out to see if the interaction could be recapitulated by using purified ERα protein. After cleaving the His6X tag from our purified MEMO1 protein, we incubated MEMO1 with His6X-tagged ligand binding domain of ERα (His6X-ERα-LBD) and performed a pull-down assay. As illustrated in **Figure 1F** , repeated washes of the beads removed excess MEMO1 (lanes 3-6) in decreasing amounts. However, elution with high levels of imidazole provided both His6X-ERα-LBD and MEMO1 indicating that there is some interaction between the two proteins. This result illustrates that MEMO1 can interact with a non-phosphorylated ERα protein that contains the peptide sequence that we used for our FP experiments.

From our biochemical and computational studies, we propose that MEMO1 interacts with ERα in a phosphorylation-independent manner at Y537. The data imply that MEMO1 may have a variety of binding modes to allow it to scaffold proteins together within signaling pathways. Our biggest question at this point is whether the peptide interaction is mimicking the biological protein-protein interaction. From the pull-down assay, it does appear that MEMO1 and the ligand-binding domain of ERα form an interaction. MEMO1 is a complex protein with multiple biological roles, and more research is necessary to understand all its functions at a molecular level (Schotanus & Van Otterloo, 2020).

## Methods

Wild-type or mutant MEMO1 were expressed in chemically competent BL21 (DE3) Star cells and purified using Ni-NTA chromatography and size exclusion chromatography as previously described (Newkirk et al., 2018). Protein purity was verified by sodium dodecyl sulfate-polyacrylamide gel electrophoresis, and the concentration was calculated form the absorbance at 280 nm and the molar extinction coefficients for wild-type or mutant MEMO1 (Newkirk et al., 2018).

Fluorescence polarization binding assays were run as previously described using 0.1 μM final concentration of the indicated fluorescein-labeled peptides (FL-pYD10, FL-ERpY, FL-ERY, FL-SRCpY, FL-SRCY) and wild-type or mutant MEMO1 at the concentrations indicated (Newkirk et al., 2018). Competitive displacement fluorescence polarization assays were performed by incubating the labeled peptide (FL-ErbB2pY1222) with wild-type MEMO1 at room temperature and adding each non-labeled competitor dilution to final concentrations of 0.1 µM and 1 µM, respectively. Each fluorescence polarization assay was pipetted in triplicate into a 96-well half-area opaque plate (90 µL/plate), including a plate blank (buffer only). Fluorescence measurements were taken using the SpectraMax i3 plate reader, and millipolarization and anisotropy were calculated. Curves of were fit with nonlinear regression in GraphPad Prism 7. Each graph is representative of at least three separate replicates performed. K _D_ values presented in the text are average ± standard deviations of at least three replicates.

Thrombin cleavage of the His6x from wild type MEMO1 was performed using a Thrombin CleanCleave ^TM^ kit following manufacturer’s protocol. The reaction was incubated at room temperature with gentle agitation for 2 hours. The protein was visualized on an SDS/PAGE gel illustrating slightly smaller molecular weight.

For the pull-down experiment, cleaved MEMO1 (0.12 mg/mL) was incubated with His6x-ERα-LBD (0.12 mg/mL) in binding buffer (50 mM sodium phosphate, pH 6.4) at 4 °C for 60 minutes with gentle shaking. HisPur beads were added and the solution was incubated at 4 °C for 60 minutes with gentle shaking. Unbound proteins were removed (flowthrough), and the beads were washed three times with wash buffer (50 mM sodium phosphate, pH 6.4, 25 mM imidazole). Bound proteins were eluted from the beads three times with elution buffer (50 mM sodium phosphate, pH 6.4, 250 mM imidazole). The samples were separated using sodium dodecyl sulfate-polyacrylamide gel electrophoresis and visualized using Coomassie blue stain.

The FL-ERpY537 and FL-ERY537 peptides were built using Schrodinger’s Maestro graphical user interface, and energy minimized using the OPLS4 force field in the TIP3P continuum solvent for water (Lu et al., 2021). The MEMO1 structure 3BCZ was obtained from the Protein Data Bank (PDB) and Chain A containing 297 residues was used after removal of the glycerol ligand. The Schrodinger Protein Preparation workflow was used on the receptor to assign bond orders, add missing hydrogens, ensure correct protonation states and convert selenomethionine to methionine residues. Schrodinger’s SiteMap was then used to predict and score potential ligand binding sites and Schrodinger’s Receptor Grid Generation was used to assemble a 30 x 40 x 20 Å receptor grid centered on each of the 3 (phosphorylated) or 4 (non-phosphorylated) highest scoring binding sites. These grids were used, along with Schrodinger’s Glide software and the SP algorithm, to dock the peptide ligands. We subjected the highest scoring poses to 4 μs of unrestrained molecular dynamics using the AMBER package with the ff14SB force field and the TIP3P water model for explicit solvation. We analyzed the resulting trajectories using cpptraj. Binding free energies, and pairwise residue interactions were approximated using MM-GBSA.

## Reagents

**Table:** 

**Peptides**		
**Name**	**Sequence**	**Source**
FL-ERpY537	FAM-NVVPLpYDLLL-NH _2_	Genscript
FL-ERY537	FAM-NVVPLYDLLL-NH _2_	Genscript
ERpY537	NVVPLpYDLLL-NH _2_	Genscript
ERY537	NVVPLYDLLL-NH _2_	Genscript
FL-SRCpY419	FAM-IEDNEpYTARQ-NH _2_	Genscript
FL-SRCY419	FAM-IEDNEYTARQ-NH _2_	Genscript
FL-ErbB2pY1222	FAM-FDNLYpYWDQD-NH _2_	Genscript
ErbB2pY1222	FDNLYpYWDQD-NH _2_	Genscript

**Other Reagents**	**Source**	
pET15b plasmid encoding WT MEMO1	Genscript	
BL21 DE3 Star Cells	Thermo Fisher Scientific	
Thrombin CleanCleave ^TM^ Kit	Sigma-Aldrich	
HisPur ^TM^ Ni-NTA Resin	Thermo Fisher Scientific	
Purified His6X-ERα ligand binding domain	Gift from Prof. Sean Fanning from Loyola University Chicago	
